# gitter: A Robust and Accurate Method for Quantification of Colony Sizes From Plate Images

**DOI:** 10.1534/g3.113.009431

**Published:** 2014-01-28

**Authors:** Omar Wagih, Leopold Parts

**Affiliations:** * European Bioinformatics Institute, Wellcome Trust Genome Campus, Hinxton CB10 1SD, UK; † Donnelly Centre for Cellular and Biomolecular Research, University of Toronto, Toronto, M5S 3E1, Canada

**Keywords:** Genetics, Image Analysis, R, Synthetic Genetic Array

## Abstract

Colony-based screens that quantify the fitness of clonal populations on solid agar plates are perhaps the most important source of genome-scale functional information in microorganisms. The images of ordered arrays of mutants produced by such experiments can be difficult to process because of laboratory-specific plate features, morphed colonies, plate edges, noise, and other artifacts. Most of the tools developed to address this problem are optimized to handle a single setup and do not work out of the box in other settings. We present gitter, an image analysis tool for robust and accurate processing of images from colony-based screens. gitter works by first finding the grid of colonies from a preprocessed image and then locating the bounds of each colony separately. We show that gitter produces comparable colony sizes to other tools in simple cases but outperforms them by being able to handle a wider variety of screens and more accurately quantify colony sizes from difficult images. gitter is freely available as an R package from http://cran.r-project.org/web/packages/gitter under the LGPL. Tutorials and demos can be found at http://omarwagih.github.io/gitter

A well-controlled approach to query the function of genes and the effects of environment or small molecules is to study single genetic perturbations in appropriate conditions. Publicly available genome-scale reagents in microorganisms, such as gene deletion collections in yeast (Tong *et al.* 2001; Giaever *et al.* 2002; Kim *et al.* 2010) and bacteria (Baba *et al.* 2006), as well as collections of tagged proteins (Ghaemmaghami *et al.* 2003; Huh *et al.* 2003), combined with low technological requirements for using them, allow almost any laboratory to conduct genome-wide studies in a comprehensive and unbiased fashion. Indeed, much has been learned about genetic interactions (Roguev *et al.* 2008; Costanzo *et al.* 2010), mechanisms of small molecule effects (Hillenmeyer *et al.* 2008), modulators of protein localization (Vizeacoumar *et al.* 2010), determinants of aging (Powers *et al.* 2006), microbial pathogenicity (Butland *et al.* 2008), and almost all other aspects of cell biology using high-throughput genetic screens.

A typical screen consists of growing an array of strains on agar plates, forming a grid of colonies on each. The raw data from a screen are a collection of high-resolution images of plates containing the colonies. A proxy for the effect of a genetic perturbation in the surveyed setting can then be estimated as the size of the corresponding colony at a particular time point or as the rate of growth over time (Shah *et al.* 2007). The primary, and perhaps the most challenging analysis task, is to accurately quantify colony sizes from these images for reliable phenotypic measurements.

Although performing the screens is accessible to many groups, the image analysis step can be a limiting factor. There are currently no tools that are able to robustly quantify the colony sizes from a wide range of experimental setups. The images of screen plates vary in format, shape, illumination, background, plate edges, typical colony sizes, and the distribution of colonies on the plate. Several existing software packages [*e.g.*, HT colony grid analyzer (Collins *et al.* 2006), Colonyzer (Lawless *et al.* 2010), Cell Profiler (Carpenter *et al.* 2006), Colony Imager (Tong *et al.* 2002), ScreenMill (Dittmar *et al.* 2010) and YeastXtract (Shah *et al.* 2007)] work well in some settings, usually for the images from the laboratory in which they were developed. However, when looking for a universal solution to a general screen analysis pipeline (Wagih *et al.* 2013), we could not find a single tool that was able to robustly handle a large variety of different plate types. Some of the tools are limited to graphical user interfaces, which makes incorporating them into new computational pipelines difficult, or require extensive customization, which is undesirable for an automated solution. Furthermore, processing irregular images, or underestimating colony sizes can be a problem, which we also demonstrate herein.

To address these issues, we developed gitter, an image analysis pipeline to robustly and accurately process images from colony-based genetic screens. gitter first applies a range of preprocessing steps, including automatic plate rotation, contrast adjustment, and background correction, followed by identification of the colony layout on the plate, by the use of techniques similar to those used for microarray image analysis (Angulo and Serra 2003; Antoniol *et al.* 2005; Berlemont *et al.* 2007; Bariamis *et al.* 2010; Rueda and Rezaeian 2011). gitter then fits the boundaries of individual colonies separately, which results in more accurate size estimates for irregular and larger-than-average colonies. We show that gitter is able to process a wide variety of plate images and is more accurate in quantifying the colony size compared to other tools. gitter can be freely obtained as an R package from CRAN under the LGPL.

## Methods

**Figure 1:**
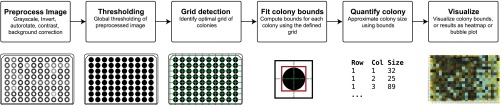
The workflow of gitter. The input image is preprocessed to account for global lighting effects and small rotations and thresholded to identify foreground pixels corresponding to colonies. The colony grid layout is fitted on the thresholded image, and individual colonies are quantified at each grid cell. Finally, the quantified colony sizes can by visualized.

There are six main steps to gitter: image preprocessing, thresholding, determining the grid of colonies on plate, identifying individual colonies, quantification of colony sizes, and visualization of the resulting data (Figure 1).

### Image preprocessing

gitter preprocesses the initial image in several steps to make it appropriate for analysis. First, the grayscale intensity *I_ij_* in row *i* and column *j* is calculated from the color image as 0.2*R_ij_* + 0.72*G_ij_* + 0.07*B_ij_*, where *R_ij_*, *G_ij_*, and *B_ij_* are the pixel intensities (ranging from 0 to 1) of the red, green, and blue channels, respectively (also known as the luminosity method). gitter assumes that colonies in the image have a greater pixel intensity compared with the background. If the colonies are dark, the user should choose to invert the image to obtain *I_ij_* = 1 − *I_ij_*. To improve running time, the fast option allows resizing images to a specified width (default 1500 pixels), while maintaining the aspect ratio.

The image is then rotated to make sure the rows of colonies are horizontal. To do so, we first calculate its Radon transform (Deans 1983), which has been successfully used in microarray image analysis (Angulo and Serra 2003; Antoniol *et al.* 2005; Berlemont *et al.* 2007; Bariamis *et al.* 2010):

$$R\left(r,\alpha \right)=\sum_{ij}I_{ij}\delta \left(r-i\cos\alpha -j\sin\alpha \right).$$

Here, *r* is the *y*-axis offset of a line from the plate center, *α* is the angle of the line, and *R*(*r*, *α*) is the sum of pixel intensities *I_ij_* along the line defined by *r* and *α*. The lines that pass through colonies and spaces between colonies will have large and small pixel intensity sums, respectively. The lines angled parallel to the rows of colonies will therefore vary between the large and small total intensity values, and *R*(*r*, *α*) will have large variance for that particular *α*. Thus, we choose the rotation angle as argmax*_α_* (var*_r_ R*(*r*, *α*)), where we vary *α* in 0.2-degree increments. To reduce the computational complexity of this step, we resize the image to a width of 500 pixels before applying the transform. In practice, images are rarely rotated more than 5 degrees because the plate position is standardized before the photo is taken. However, we allow rotation angles of up to 30 degrees.

Artifacts such as variation in lighting, condensation, and noise can make it difficult to distinguish colonies from the plate. We estimate the background by eroding and then dilating the image using a *w* × *w* window and then subtract it from original image to remove the broad artifacts. We picked the window size *w* as 1.5 times the width of the image divided by the number of colony columns in the plate. This ensures colonies and plate edges are smaller than the window, and will be collapsed in the erosion step.

### Thresholding the image

Next, the image is thresholded to identify foreground pixels corresponding to colonies and other bright objects in the plate. We use k-means clustering with two clusters to find the threshold value *t* that distinguishes the two classes. We initialize *t* to the mean of *I_ij_*, and then iteratively recalculate cluster means as the average intensity of pixels above and below *t*, respectively, and threshold *t* as the midpoint of cluster means. These steps are repeated until the value of *t* does not change.

### Finding the grid of colonies

**Figure 2:**
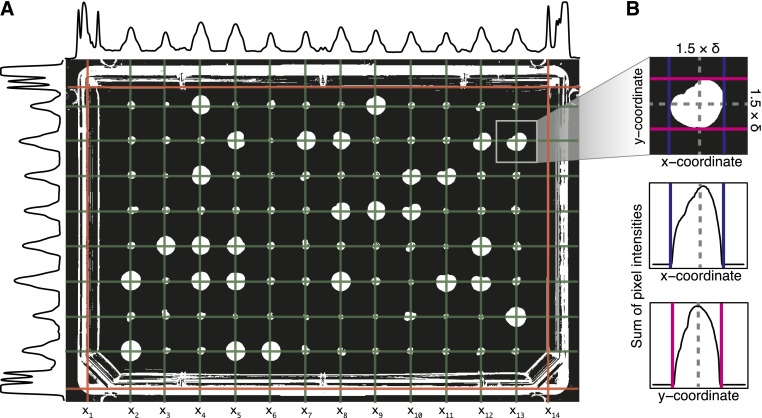
Obtaining the grid of colonies. (A) Total pixel intensities *T_i_* for rows (to the left of image) and columns (above image) of the thresholded input image are used to find the centers of the peaks that correspond to colony locations (green lines) that define the colony grid. Peaks from greater pixel intensities near plate edges and other sources of confounding signals (red lines) are discarded by fitting the grid. (B) Identifying the best-fit boundaries for a spot using local intensity profiles. Magenta and blue lines represent the x and y-coordinates of the boundaries, respectively. Gray dashed lines represent the local maxima from (A) that define the colony center.

In an image of a screen plate, pixel intensities in rows and columns corresponding to the colonies tend to have greater values than elsewhere (Figure 2A). This feature can be used to determine the colony locations. To do so, we first calculate the total number of foreground pixels in row *i* as $T_{i}=\sum_{j}\text{I}\left[I_{ij}\geq t\right]$, yielding a set of characteristic peaks. Some of these peaks correspond to colonies (Figure 2A, green lines), rest to plate edges or other artifacts (Figure 2A, red lines). The number of foreground pixels in the rows corresponding to one round colony is expected to be $\lambda_{w}=2\sqrt{W^{2}-w^{2}}$, where *w* ranges from −*W* to *W*, the colony radius. To quantify how well the intensity profiles match the expectation at each potential colony center *i*, we compute the Pearson correlation *r_i_* between the expected intensity *λ* = (*λ*_−_*_W_*,…,*λ_W_*) and the observed intensities in the window (*T_i_*_−_*_W_*,…,*T_i_*_+_*_W_*) centered on *i*. Here, *W* is estimated as half the median distance between local maxima of the middle 50% in smoothed intensity profiles. We determine all *N* high peaks in the correlation profiles by finding the local maxima $\left\{x_{k}|r_{x_{k}}>\text{max}\left(r_{x_{k-1}},r_{x_{k+1}},0.3\right)\right\}$, and calculate the distance *δ_k_* = *x_k_* − *x_k_*_−1_ between them. Given the start index of the true colony peaks *s*, and the number of colonies, *n* in the row, we then calculate the likelihood *L*(*s*) for different starting positions, assuming normal distributions for distances *δ_k_* between the peaks, and the correlations to expected colony shape *r_i_* at the peak centers:

$$\begin{array}{c} L\left(s\right)=P\left(x|s;\mu_{\delta },\sigma_{\delta }^{2}\right)P\left(r|s;\mu_{r},\sigma_{r}^{2}\right)\\ =\prod_{k=s}^{s+n-2}N\left(x_{k+1}-x_{k};\mu_{\delta },\sigma_{\delta }^{2}\right)\times \\ \times \prod_{k=s}^{s+n-1}N\left(r_{x_{k}};\mu_{r},\sigma_{r}^{2}\right). \end{array}$$

Here, $\mu_{\delta }=\frac{\sum_{k}\delta_{k}}{N-1}$, $\sigma_{\delta }^{2}=\text{var}\left(\delta_{k}\right)$, $\mu_{r}=\frac{\sum_{k}r_{x_{k}}}{N}$, and $\sigma_{r}^{2}=\text{var}\left(r_{x_{k}}\right)$. We pick the most likely starting peak $\hat{s}={\text{argmax}}_{s}L\left(s\right)$, and the corresponding true peaks $\left(x_{\hat{s}},\ldots ,x_{\hat{s}+n-1}\right)$ that follow. The aforementioned procedure is repeated similarly for columns and true peaks obtained from rows and columns are used to define the initial colony locations for the next analysis step. We also calculate the characteristic distance between true peaks $\delta =\text{median}\left(\delta_{x_{\hat{s}}},\ldots ,\delta_{x_{\hat{s}+n-2}}\right)$.

### Quantification of colony size

First, small speckles are optionally removed from the thresholded image by eroding with a 3 × 3 window, which eliminates single pixel noise. Larger windows would start eroding colonies and substantially reduce their measured size. We then find, for each colony, the minimum rectangle enclosing it in the square of width 1.5 × *δ* around the colony center. This width is chosen to allow for variations in the colony’s morphology outside its typical boundary. We then identify the local minimum of the colony pixel intensity profiles that are nearest to the colony centers, but at least $\frac{\delta }{3}$ away (Figure 2B). If the pixel defining the center of the colony is a background pixel, we assume that the colony is not present, and a square with width *δ* around the center of the colony is used as the bounding rectangle. Finally, the size or area *A* of the colony is computed as the number of foreground pixels within the boundary. For each colony, we also report its circularity $4\pi \frac{A}{P^{2}}$, where *P* is the number of foreground pixels neighboring a background pixel.

If the boundary contains foreground pixels, there is no clean separation between neighboring colonies, suggesting they are at least touching. In this case, the colony is flagged as potentially overlapping in the output. Similarly, colonies with low circularity are flagged to draw users’ attention to them, and allow automatic filtering. Entire plates are flagged if at least 10% of colonies are smaller than 0.1 of the median colony size, or have circularity below 0.6. Plate level flags indicate to the user that the output should be manually inspected.

### Processing sparse images

Sparse plates with a majority of small or dead colonies, such as those seen in suppression screens, usually fail to process properly, as there is not enough information to establish the grid. To overcome this, the typical distance between colonies *δ* and colony center coordinates {*x_k_*} can be precomputed from a reference image taken with same dimensions, sizes and settings as the sparse image. To account for small translational shifts, we calculate total pixel intensities *T_i_* for both the reference and the sparse image, and offset the reference image to maximize the correlation between the *T_i_* profiles. After obtaining the colony center coordinates and typical distances between them from the reference, the sparse image can be processed without fitting its grid independently.

## Results

gitter was developed to overcome the two main drawbacks of existing tools—limited range of experimental setups that can be easily analyzed, and accuracy in quantifying the colony sizes in nontrivial cases. Thus, we evaluated the performance of gitter for robustness of handling plates from different laboratories, and correctly estimating the size of colonies.

### Robustness

We tested gitter on images from a range of experimental setups and organisms, including 840 images from *Saccharomyces*** ***cerevisiae* (Costanzo *et al.* 2010), 82 from *Schizosaccharomyces pombe*, 87 from *Escherichia coli* (Butland *et al.* 2008; Babu *et al.* 2011) and nine from *Drosophila melanogaster* (Hens *et al.* 2011). All *D. melanogaster* and *E. coli* images were successfully gridded and quantified without any adjustments to the default parameters. Only 2 of 87 (2.3%) of *S. pombe* images and 5 of 840 (0.6%) *S. cerevisiae* images had an edge being mistaken for a row or column. When processing images in batch using a reference image, all tested images were correctly gridded.

**Figure 3:**
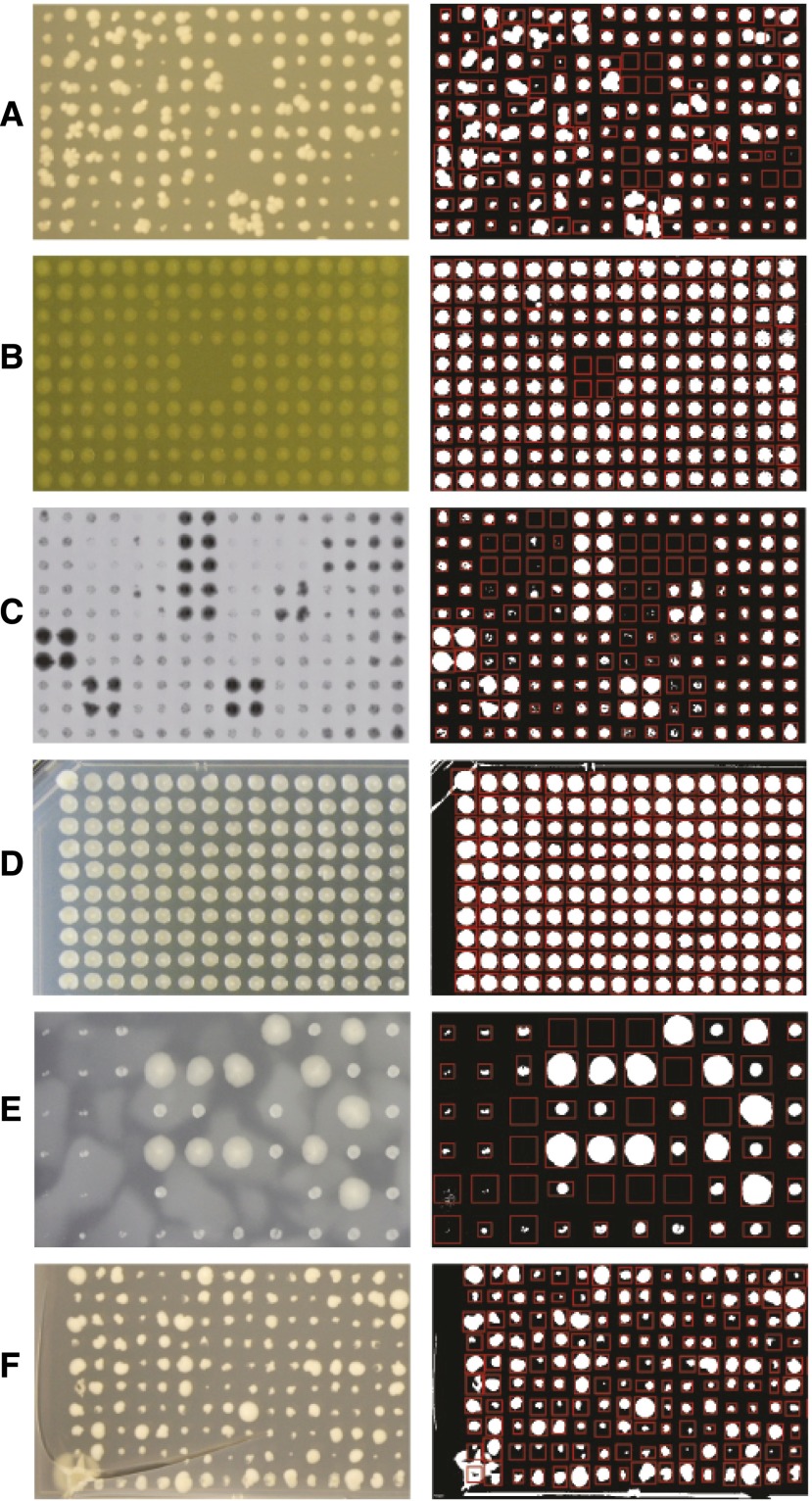
Sections of five example images and their thresholded gridded output. gitter can quantify images with (A) irregular colonies, (B) noise, (C) inverted colonies, (D) variable illumination, (E) cloudy background, and (F) cracked plates.

The tested images included many that pose difficulties to existing image analysis tools. The sizes of irregular colonies (Figure 3A) are difficult to quantify accurately by counting pixels inside a fixed circular template, but gitter overcomes this problem by fitting the bounds of each colony separately. gitter can also process noisy and inverted images where colonies are represented by darker, rather than brighter colors (Figure 3, B and C). Images with uneven lighting or cloudiness caused by condensation (Figure 3, D and E) are problematic because brighter regions often are mistaken for foreground pixels. gitter’s background correction subtracts these effects out so they do not interfere with gridding and colony quantification. Images of plates with cracked agar caused by drying, dropping, etc. (Figure 3F) often are discarded because it is assumed that the image analysis software will not be able to successfully identify the colonies from the deformed grid. gitter overcomes small variations in the grid by refitting individual colony bounds.

### Accuracy

We compared the results of gitter to Colonyzer (Lawless *et al.* 2010), HT colony grid analyzer (Collins *et al.* 2006), and Colony Imager (Tong *et al.* 2002). Three images showing irregular colonies and that were successfully processed by the four tools were considered for the comparison. Establishing a ground truth segmentation and boundary for colonies is difficult because thresholding is subjective. We thus first confirmed that the colony size estimates are concordant for a large fraction of colonies, and then manually inspected discordant ones.

**Figure 4:**
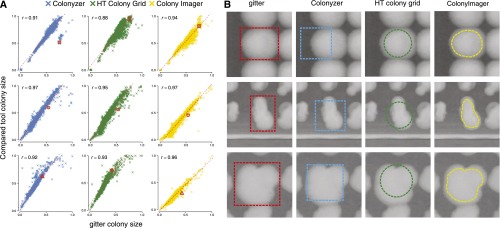
Comparison of gitter and other tools. (A) Comparison of colony sizes calculated by the four compared tools for three irregular images that pass each tool. Colony sizes are normalized to a range of 0−1 by dividing by the maximum colony size. (B) Fitted colony bounds for the four compared tools for three colonies. The dashed red boundary designates the region of the colony that is used for quantification. Sizes of these colonies are marked on the scatter plots (A) with a red marker.

The colony sizes calculated by different tools are similar for well-behaved images and colonies (Figure 4A), but the estimates differ for some classes. Although Colonyzer also optimizes the bounds of each spot, it does so by fitting a fixed width square, resulting in underestimating sizes of spots larger than the square (Figure 4B). In addition, in some rare cases, the bounds are misplaced for colonies on the edge of a plate (Figure 4B). HT colony grid fits a grid of fixed-size circles to the image. Due to not adjusting the bounds for each colony, the sizes are sometimes underestimated, as a substantial portion of the colony remains outside of the fixed circle (Figure 4B). Colony Imager approximates the edges of each spot; however, we have found the defined bounds to be eroded (Figure 4B), resulting in the underestimation of colony sizes. This becomes an issue for very small colonies, as they get a quantified size of zero, and very large colonies, for which the erosion can account for a large part of the total area.

## Implementation

gitter is implemented in an open-source R package available from http://cran.r-project.org/web/packages/gitter and uses the EBImage (Pau *et al.* 2010) package for image manipulation and the ggplot2 (Wickham 2009) package for visualizations. A single function call quantifies the colony sizes from an image:

gitter(file, plate.format, remove.noise=F,

inverse=F, autorotate=F,

contrast=F, fast=1500, verbose=p)

where file is the path to the plate image, plate.format is a length-2 vector of the number of rows and columns of colonies in the plate or a known plate density (*e.g.*, 1536 or 384), remove.noise is a boolean determining whether noise and speckles should be removed before quantification, inverse determines whether the image should be inverted, autorotate determines whether the image should be autorotated, contrast is a contrast factor (if any), that should be applied to the image, fast is the width (in pixels) that the image should be resized to before processing, and verbose indicates whether the progress should be written out to the console. A set of images can be processed in batch using the batch function:

gitter.batch(files, file.reference, …)

where files is the directory containing images to be processed or a list of file paths, file.reference is the path to a reference image to process all images in file (optional), and … is any of the previously mentioned arguments. Additional documentation can be found online through the documentation on the package website.

The result of calling gitter on a single file is a data frame containing the row and column of the colony on the plate, the quantified size, circularity, and any warning flags (potential overlap with neighbor and low circularity). This data frame is output to a tab-delimited file, and the thresholded image showing the boundaries of each colony (as in Figure 3B) is created. Users can also visualize the quantified colony sizes as a heatmap or bubble plot.

## Discussion

Software for analyzing images of genetic screens does not always work out of the box for a new experimental setup. Here, we present gitter, a tool that provides a robust and accurate way to quantify colony sizes via a simple interface. We apply methods to deal with heterogeneity in plate formats, as well as confounding factors such as rotation and illumination differences, colony morphology, and plate edges. As a result, the vast majority of the tested images can be processed without requiring any input from the user to tweak the parameters, and all tested images are successfully processed in batch provided one well-behaving reference.

gitter optimizes the boundaries for each colony separately, resulting in more accurate colony size estimates. This is especially beneficial for screens with irregular or larger-than-average colonies, and we have shown that comparable tools often underestimate colony sizes in such cases. As the downstream analyses look for the effects of genetic perturbations by comparing colony sizes, these minute differences in estimates can have a substantial impact on the biological interpretation of the screen.

The image analysis approach used by gitter required choosing several algorithms and variables *a priori*. For example, we used the Radon transform as a natural approach to quantify the variation in different directions of the image. We also tested the 2-dimensional Fourier transform but found extracting the optimal rotation angle from it to be more error-prone. The 3 × 3 kernel was used for noise removal as the smallest that eliminates one pixel speckles, since larger kernels start compromising colony size by erosion. We picked the background correction window size *w* to be larger than a colony diameter so that erosion and dilation remove the foreground objects. The choice of even-larger *w* results in further blurring of the signal and reduction of correction efficacy, whereas choosing a much smaller *w* results in subtracting off parts of colonies. The typical width *W* is estimated from the central 50% of intensity profiles to include only well-behaved portions of the image, and exclude edges. Finally, we used a window of 1.5 × *δ* around colony centers to search for the optimal boundary while allowing for variations in the colony’s morphology. Smaller windows produce boundaries that cut off parts of irregular colonies, and larger ones start encompassing neighboring colonies. All these parameters were manually optimized for best performance on a large set of test images.

Automated tools for extracting understanding from biological data ought to be powerful and flexible on one hand, but intuitive and approachable for bench scientists on the other. Although to use gitter requires one to have a basic knowledge of R, we have also made it accessible via the online SGAtools normalization, scoring, and visualization suite (Wagih *et al.* 2013), so that images can be processed in a web application without a command line interface. gitter’s output is compatible with standard formats used in colony processing software, allowing integration into existing data analysis pipelines (Bean and Ideker 2012; Wagih *et al.* 2013). Future work includes adding support for spot dilutions, tetrad plates, densitometric images, and multiplate images; the latter can currently be processed with other existing tools (Dittmar *et al.* 2010; Lawless *et al.* 2010). We hope that with gitter and SGAtools the transition from images of a screen to understanding the effects of the perturbation is straightforward for experimentalists and not limited by computation.

## Supplementary Material

Supporting Information
