# Supplementary material for: gitter: A Robust and Accurate Method for Quantification of Colony Sizes From Plate Images
Source: G3 (Bethesda). 2014 Jan 28;4(3):547–52. doi: 10.1534/g3.113.009431 (PMC3962492; doi:10.1534/g3.113.009431)
Supplement: Supporting Information [file supp_4_3_547__index.html]

gitter: A Robust and Accurate Method for Quantification of Colony Sizes from Plate Images — gitter: A Robust and Accurate Method for Quantification of Colony Sizes From Plate Images — Supporting Information 

# gitter: A Robust and Accurate Method for Quantification of Colony Sizes From Plate Images

## Supporting Information for Wagih and Parts, 2014

gitter is freely available for download as an R package at http://cran.r-project.org/web/packages/gitter. Tutorials and demos can be found at http://omarwagih.github.io/gitter.
